# Point-of-care C-reactive protein to assist in primary care management of children with suspected non-serious lower respiratory tract infection: a randomised controlled trial

**DOI:** 10.3399/bjgpopen18X101600

**Published:** 2018-07-11

**Authors:** Marjolein JC Schot, Ann Van den Bruel, Berna DL Broekhuizen, Jochen WL Cals, Eveline A Noteboom, Walter Balemans, Rogier M Hopstaken, Sanne van Delft, Niek J de Wit, Theo JM Verheij

**Affiliations:** 1 GP and PhD Student, Julius Center for Health Sciences and Primary Care, University Medical Center Utrecht, Utrecht, Netherlands; 2 GP and Associate Professor, Department of Primary Care Health Sciences, University of Oxford, Oxford, UK; 3 GP, General Practice de Bongerd, Borculo, Netherlands; 4 GP and Professor of Primary Care, Department of Family Medicine, CAPHRI School for Public Health and Primary Care, Maastricht University, Maastricht, Netherlands; 5 PhD Student, Julius Center for Health Sciences and Primary Care, University Medical Center, Utrecht, Netherlands; 6 GP and Innovation Specialist, Star-SHL, Etten-Leur, Netherlands; 7 Manager of Innovation, Saltro Diagnostic Center for Primary Care, Utrecht, Netherlands; 8 GP and Professor of Primary Care, Julius Center for Health Sciences and Primary Care, University Medical Center Utrecht, Utrecht, Netherlands; 9 GP and Professor of Primary Care, Julius Center for Health Sciences and Primary Care, University Medical Center Utrecht, Utrecht, Netherlands

**Keywords:** Point-of-care CRP testing, lower respiratory tract infection, children, general practice, c reactive protein

## Abstract

**Background:**

Overprescription of antibiotics for lower respiratory tract infections (LRTIs) in children is common, partly due to diagnostic uncertainty, in which case the addition of point-of-care (POC) C-reactive protein (CRP) testing can be of aid.

**Aim:**

To assess whether use of POC CRP by the GP reduces antibiotic prescriptions in children with suspected non-serious LRTI.

**Design & setting:**

An open, pragmatic, randomised controlled trial in daytime general practice and out-of-hours services.

**Method:**

Children between 3 months and 12 years of age with acute cough and fever were included and randomised to either use of POC CRP or usual care. Antibiotic prescription rates were measured and compared between groups using generalising estimating equations.

**Results:**

There was no statistically significant reduction in antibiotic prescriptions in the GP use of CRP group (30.9% versus 39.4%; odds ratio [OR] 0.6; 95% confidence interval [CI] = 0.29 to 1.23). Only the estimated severity of illness was related to antibiotic prescription. Forty-six per cent of children had POC CRP levels <10mg/L.

**Conclusion:**

It is still uncertain whether POC CRP measurement in children with non-serious respiratory tract infection presenting to general practice can reduce the prescription of antibiotics. Until new research provides further evidence, POC CRP measurement in these children is not recommended.

## How this fits in

POC CRP testing has added value in the diagnosis of pneumonia in adults, and has proven to safely reduce antibiotic prescriptions in general practice. In children, POC CRP testing has proven valuable in ruling out serious infections, but the effect of its use by GPs on the prescription of antibiotics was not known. There was no significant reduction in antibiotic prescriptions when GPs used POC CRP testing in children with suspected non-serious LRTI.

## Introduction

Acute respiratory tract infections are the most common diagnoses in children in primary care.^1,2^ Childhood LRTIs include acute bronchitis, bronchiolitis, and pneumonia. Pneumonia is a rare but serious condition and should be treated with antibiotics because of the difficulty in distinguishing viral from bacterial causes,^3,4^ whereas bronchitis and bronchiolitis are more common and usually self-limiting illnesses.^5–7^

Despite being of value in only a minority of children with LRTI, and contrary to recommendations in national guidelines,^4^ antibiotics are frequently prescribed in general practice in the Netherlands, with prescription rates varying between 56 and 70%.^1,8,9^ Diagnostic uncertainty, parental worries and expectations, or the GP’s anticipation of these, are important drivers of antibiotic prescriptions.^8,10,11^ Even in a low prescribing country like the Netherlands, 48–63% of antibiotic prescriptions are thought to be inappropriate.^10,12^ This is harmful as antibiotics cause side effects,^12^ increase re-consultation rates,^13^ and contribute to antimicrobial resistance. Repeated use of antibiotics increases antimicrobial resistance in communities but also in individuals,^14–15^ making it important to correctly identify children who need antibiotics, but equally important to protect those who will not benefit.

Although CRP levels do not allow differentiation between bacterial or viral origin of an infection in adults or children, they are proxy for the disease severity.^16–18^ In adults, POC CRP has added value in the diagnosis of pneumonia^19–21^ and safely reduces antibiotic prescriptions for acute respiratory tract infections in primary care.^22^ Following this evidence, national guidelines on acute cough recommend POC CRP testing for adults in case of diagnostic uncertainty,^4^ similar to the current National Institute for Health and Care Excellence guideline on pneumonia in adults.^23^ More than half of all Dutch GPs now have access to POC CRP testing, in daytime practice as well as at out-of-hours services.^24–25^

Although POC CRP is also of diagnostic value for diagnosing pneumonia in children^26^ and useful in ruling out serious infection in children,^27^ its effect on antibiotic prescribing for children with symptoms of LRTI is unclear. In this study, it was assessed whether POC CRP testing in children with a suspected non-serious LRTI reduces antibiotic prescribing compared to usual care without CRP testing.

## Method

This is a pragmatic, open, randomised controlled two-arm trial in primary care.

### Participants and setting

Between December 2013 and May 2016, children aged between 3 months and 12 years were recruited in 28 daytime general practices across three different regions in the Netherlands. Due to slow recruitment rates, children were additionally recruited at four out-of-hours services between November 2015 and May 2016. Children were eligible for inclusion if they had acute cough, reported fever, and were suspected of having a non-serious LRTI by the treating GP. Children who were judged as severely ill or highly suspect of pneumonia were excluded (Box 1). Parents provided written informed consent.

**Box 1.** Eligibility criteria

**Inclusion (all criteria must be present)**	**Exclusion (any presence of)**
Suspicion of lower respiratory tract infection	Impaired immunity
Age 3 months–12 years	Severe pulmonary disease
Acute cough <21 days	Serious congenital defects
Reported fever >38 °C, <5 days	Use of systemic antibiotics and/or corticosteroids in past 4 weeks
	Judged severely ill by the GP based on symptoms and signs
	Highly suspected of having pneumonia by the GP
	Referral to specialist or emergency department deemed necessary by GP

### Randomisation

Daytime general practices were cluster randomised per practice, to avoid contamination. Furthermore, it was that expected GPs might experience a learning effect from conducting CRP tests. By linking CRP levels to apparent severity of illness, this could have affected prescribing in the control group. Block randomisation stratified by region and practice type (academic versus non-academic) was used.^28^

Arguments for cluster randomisation were not applicable to an out-of-hours service, where GPs participated in this study during one shift, and included two children at most. Therefore, children recruited at out-of-hours services were individually randomised using sequentially numbered opaque sealed envelopes (SNOSE).^29^ The SNOSE piles were prepared by a member of the research team, using permuted block randomisation. After the treating GP checked eligibility, an onsite research assistant, blinded to the clinical evaluation of the child, opened the envelope.

### Intervention

For children in the intervention group, a POC CRP test was performed after clinical assessment by the treating GP. In the control group GPs were advised not to use POC CRP, and treatment decisions were based on clinical assessment as usual.

CRP was measured using an Afinion^™^ POC testing device (Alere Technologies AS, Oslo, Norway), with a measurement range between 5 and 160 mg/L, and reliable for use in children.^30^ The result of the test is available within 4 minutes, requiring 1.5 μL of blood obtained via finger prick.

GPs were not provided with strict decision rules based on CRP levels, but were given the following guidance:

1. POC CRP levels should be interpreted in combination with symptoms and signs.
2. CRP levels <10mg/L make pneumonia less likely, but should not be used to exclude pneumonia when the GP finds the child ill, or when duration of symptoms is <6 hours.
3. CRP levels >100mg/L make pneumonia much more likely, however, such levels can also be caused by viral infections.
4. Between 10mg/L and 100mg/L, the likelihood of pneumonia increases with increasing CRP levels.

All management decisions including the use of other diagnostic tests and treatment were left to the GP’s discretion.

### Data collection

At baseline, GPs recorded the child’s temperature and assessed illness severity on a Visual Analogue Scale (VAS). At the end of consultation they registered their working diagnosis and treatment plan. Three months after inclusion, children's medical records were reviewed to collect data on secondary outcomes.

### Outcomes

The primary outcome was antibiotic prescribing at the index consultation. Secondary outcomes were re-consultation and antibiotic prescribing during the same illness episode, consultation for a new episode of any respiratory tract infection within 3 months of the index consultation, and antibiotic prescriptions at these consultations.

### Sample size calculation

Sample size calculation was based on the expectation that POC CRP testing would reduce antibiotic prescribing by at least 20%, from 70% to 50%. To detect such a difference with 80% power and two-sided 5% significance, and considering a cluster size of 16 and an intracluster correlation coefficient of 0.06, a total of 354 patients were required. After expanding recruitment to the out-of-hours services the sample size calculations were not altered as a cluster effect is not present for the children individually randomised at the out-of-hours services and this would most likely lead to a reduction in the number of children needed.

### Statistical analysis

For the primary outcome, data were analysed with an intention-to-treat approach using general estimating equations to account for cluster effects and the baseline characteristics age, estimated illness severity, inclusion at out-of-hours service, and index of deprivation based on postal code. Children with missing outcomes were excluded from the analysis. Additionally, the primary outcome was analysed using a per protocol approach. Secondary outcomes are analysed using generalised estimating equations to account for cluster effects. Analysis was done using SPSS (version 21).

## Results

**Figure 1.**
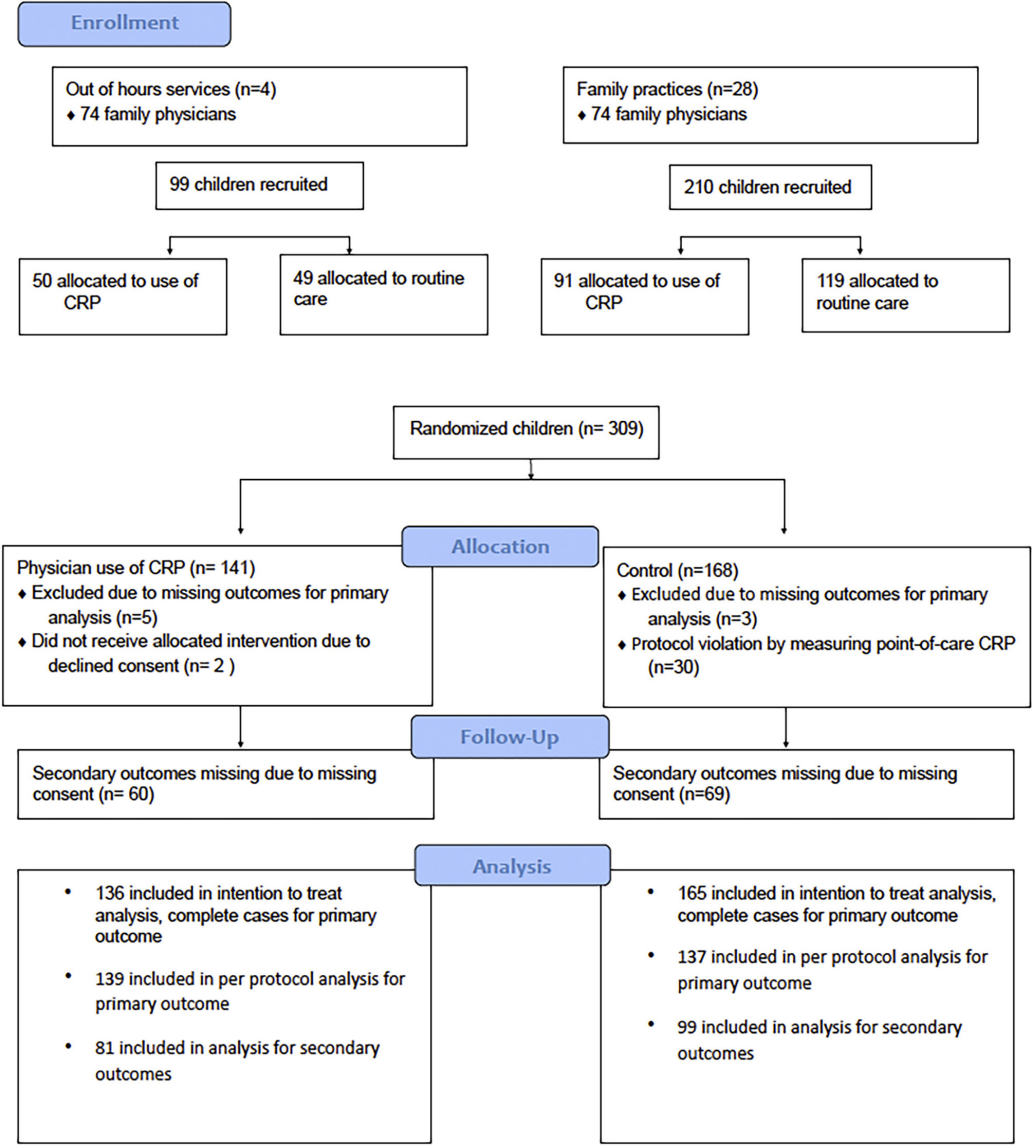
Trial profile

Three hundred and nine children were recruited by 148 GPs (Figure 1). Eight children were excluded due to missing age (*n *= 7) or severity of illness score (*n *=1) at baseline. Characteristics of children in both groups were similar regarding age, sex, symptoms, fever, and estimated illness severity. In the control group, more children had a low social economic status (Table 1). GPs noted bronchitis as their final diagnosis in 21.3% of the children, and pneumonia in 13.3%. All diagnoses are listed in Table 2. Based on estimated illness severity, children presenting to the out-of-hours service were not more severely ill than children presenting to daytime general practice (mean VAS score 3.7 versus 4.0; *P *= 0.2).

**Table 1.** Characteristics of randomised children at baseline

	GP use of CRP (*N *= 136)	Control (*N *= 165)
Median age, years (range)	3 (0–11)	2 (0–11)
Female sex, *n* (%)	65 (47.8)	81 (49.1)
Abnormalities at auscultation, *n* (%)	71 (50.4)	83 (49.4)
Signs of otitis media acuta, *n* (%)	13 (9.2)	23 (13.7)
Signs of tonsillitis, *n* (%)	17 (12.1)	18 (10.7)
Mean temperature, °C	38.2	38.0
Estimated severity of illness by GP, range (mean)	0.3–8.5 (4.0)	0–8.0 (3.8)
Recruited at out-of-hours service	49 (36.0)	49 (29.7)
Low social economic status	4 (2.9)	17 (10.3)

**Table 2.** Recorded diagnosis by GP after medical history, physical examination, and point-of-care C-reactive protein if applicable (*N* = 301)

Diagnosis	*n*	%
Upper respiratory tract infection	93	30.9
Bronchitis	64	21.3
Pneumonia	40	13.3
Cough	27	9
Viral respiratory tract infection	14	4.7
Influenza	12	4
Fever	11	3.7
Bronchial hyperreactivity	9	3
Otitis media acuta	9	3
Lower respiratory tract infection	7	2.3
Respiratory tract infection, not specified	7	2.3
Acute laryngitis or tracheitis	1	0.3
Otitis media with effusion	1	0.3
No diagnosis noted	6	2

### Antibiotic prescription and re-consultations

GPs in the CRP group prescribed antibiotics to 30.9% of the children compared to 39.4% in the control group (OR 0.6; 95% CI = 0.30 to 1.24). The only factor significantly related to the prescription of antibiotics was the estimated illness severity (OR 1.44; 95% CI = 1.26 to 1.66).

POC CRP was not measured in two children in the intervention group (1.4%), and in the control group point-of care CRP was measured 30 times (18.2%), in violation of protocol (Figure 1). A per protocol analysis, excluding these 32 children, showed no significant difference in antibiotic prescription rates at the index consultation.

Due to missing consent of the parents for follow-up, follow-up data were only available for 180 children (58% of total, 81/141 children [57%] in the intervention group and 99/168 children [59%] in control group). Children in both groups had similar rates of re-consultations within the same episode of illness (33% versus 34%; OR 0.95; 95% CI = 0.46 to 1.99) and antibiotic prescriptions during these consultations (7% versus 8%; OR 0.94; 95% CI = 0.33 to 2.63). In the next 3 months, 16% of children in the CRP group consulted their GP for respiratory tract illness, compared to 29% in the control group (OR 0.61; 95% CI = 0.32 to 1.17) (Table 3). One child in the control group was admitted to hospital directly after inclusion.

**Table 3.** Effects of CRP testing on secondary outcomes

	GP use of CRP (*N *= 81) *n* (%)	Control (*N *= 99) *n* (%)	Odds ratio (95% CI)
Re-consultation for baseline episode of illness	27 (33)	34 (34)	0.95 (0.46 to1.99)
Antibiotics for baseline episode of illness	6 (7)	8 (8)	0.94 (0.33 to 2.63)
Non-urgent referral to secondary care for baseline episode of illness	3 (4)	5 (5)	0.93 (0.18 to 4.86)
Consultation for new episode of RTI within 3 months	13 (16)	29 (29)	0.61 (0.32 to 1.17)
Antibiotics for new episode of RTI within 3 months	2 (2)	7 (7)	0.34 (0.08 to 1.39)
Non-urgent referral to secondary care for new episode of RTI	3 (4)	7 (7)	0.54 (0.10 to 2.79)

CI = confidence interval. RTI = respiratory tract infection.

### CRP levels and antibiotic prescriptions

CRP levels ranged from <5 to 200 mg/L, with 46% of children having a CRP level <10 mg/L, 51% 10–100 mg/L, and 4% >100 mg/L. Control children in whom CRP was measured were not more seriously ill than other control children (mean illness severity 3.8 in both groups; *P *= 0.9), and their mean CRP level was not significantly different from the mean CRP level in children in the intervention group (mean 22.5 versus 24.9; *P *= 0.72).

**Figure 2.**
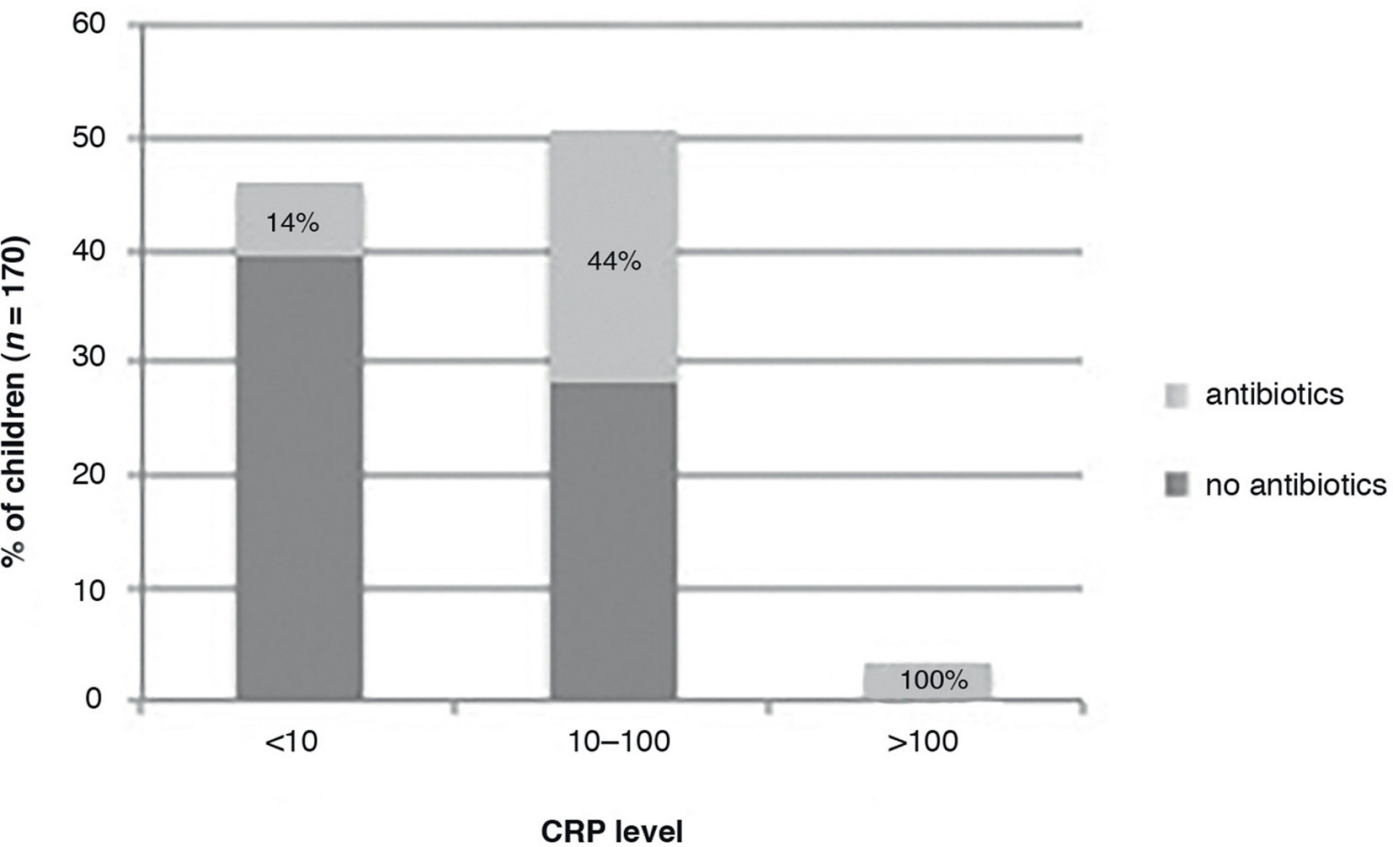
CRP levels and antibiotic prescriptions.

Children were more likely to get an antibiotic prescription with increasing CRP level, ranging from 14% in children with a CRP level <10 mg/L to more than 50% in children with a CRP level >40 mg/L (Figure 2).

## Discussion

### Summary

In this open, pragmatic, randomised controlled trial in primary care, there was no significant effect on antibiotic prescribing for children with non-serious respiratory tract infection when GPs used POC CRP: antibiotic prescribing was 30.9% in the CRP group versus 39.4% in the intervention group. Re-consultation and antibiotic prescriptions in the following 3 months also did not differ significantly.

### Strengths and weaknesses

A pragmatic design was used, evaluating POC CRP testing in daytime general practices and out-of-hours services, making the results generalisable to routine general practice. The cluster design in daytime general practices aimed to minimise learning effects and contamination. Nevertheless, there were protocol violations in the control group, potentially diluting the effect of CRP. A per protocol analysis, however, did not find a significant effect.

Although a trend towards reduction in antibiotic prescriptions was observed, it was not possible to prove that this is statistically significant. This may in part be due to lack of power to detect this smaller than expected decrease. Antibiotic prescribing rates were lower than expected in both groups. Based on earlier studies in children with LRTI, the authors presumed an antibiotic prescription rate of 70% in the control group in the sample size calculation.^1,9^ This lower prescription rate may have been caused by the recruited patient mix, in particular by the inclusion of children with an upper respiratory tract infection in whom antibiotics are known to be prescribed less frequently.^10–11,31^ Although this study aimed to include children with LRTI and the inclusion criteria were designed accordingly, there is a discrepancy between these criteria and the GP’s reported diagnosis after complete assessment. Often a symptom-based diagnosis, or an upper respiratory tract infection was reported. The open character of this study, with the GP unblinded to the CRP level before noting a final diagnosis, might have influenced diagnostic labelling.

The study did not reach the planned sample size (309 out of 354 children), despite a prolonged recruitment period and addition of the out-of-hours services for recruitment. This may have further affected the power of the study, and a larger study is necessary to decide whether POC CRP can reduce antibiotic prescriptions. Although the study aimed for a large reduction in the prescription of antibiotics based on results from trials in adults,^32^ if a future study could confirm these results, a decrease of 8.5% in antibiotic prescriptions in this group of primary care patients could be considered clinically relevant, as in other studies with the same aim in adults.^33–34^

Data for analysis of secondary outcomes were available for 58% of the children. Quite low follow-up rates were the result of a need for obligatory double consent in the Netherlands, signed by both parents, to collect follow-up. This double consent proved difficult to obtain in general practice.^35^ There is no indication that loss to follow-up was related to any other factors.

### Comparison with existing literature

CRP levels in this study correspond with reported levels in adults^32^ and children with respiratory infections.^27,36^ Most children had low CRP levels, as is expected in a primary care setting, because most children suffer from non-serious illnesses. In a recent Norwegian study in children with fever and/or respiratory symptoms presenting to out-of-hours services, antibiotics were prescribed to 13% of children with CRP levels <20 mg/L.^37^ In the current study, 14% of children with a CRP level <10 mg/L were prescribed antibiotics. As in the current study, a CRP level >20 mg/L was found to be a strong predictor for the prescription of antibiotics.

### Implications for research and practice

It is still uncertain whether POC CRP can reduce antibiotic prescriptions for children with suspected non-serious LRTI. Future research should focus on this question.

Future research should also focus on the value that POC CRP potentially has in more correctly identifying the children in primary care that suffer from pneumonia, as current evidence shows no definite cut-off levels that are useful to rule in the child in need of antibiotic treatment. This could lead to uncertainty in management of children with intermediate to higher CRP levels.

More than half of children with a POC CRP level >40 mg/L in this study were prescribed antibiotics. CRP POC testing was introduced in primary care for adults with LRTI, to support decisions on antibiotic prescribing.^22^ This may have led GPs to consider elevated CRP levels as a proxy for bacterial infection automatically warranting antibiotics. However, CRP levels do not allow differentiation between bacterial or viral origin of infection, but are a proxy for the disease severity.^16–18^ Therefore, an elevated CRP level in children is a red flag for potential serious infection. This may require treatment with antibiotics, but should especially prompt the GP to ensure proper instruction of parents and careful safety-netting. Efforts should be made to educate GPs on the current evidence on the value of POC CRP for children. Further research is necessary to provide them with threshold-specific recommendations.

In this study, children with low CRP levels were prescribed antibiotics, although children who were judged as severely ill or highly suspected of having pneumonia were excluded, and despite evidence that CRP levels <5 mg/L can safely rule out serious infections requiring hospitalisation in children.^27^ Knowledge on the GPs’ reasoning behind these prescriptions, including possible non-medical reasons, might provide further insights to better target interventions for antibiotic stewardship.

In conclusion, it is still uncertain whether POC CRP measurement in children with non-serious respiratory tract infection presenting to general practice can reduce the prescription of antibiotics. Until further research provides more evidence, POC CRP measurement in children with non-serious respiratory tract infection is not recommended.
